# The Astronomical Orientation of Ancient Greek Temples

**DOI:** 10.1371/journal.pone.0007903

**Published:** 2009-11-19

**Authors:** Alun M. Salt

**Affiliations:** School of Archaeology and Ancient History, University of Leicester, Leicester, United Kingdom; University of East Piedmont, Italy

## Abstract

Despite its appearing to be a simple question to answer, there has been no consensus as to whether or not the alignments of ancient Greek temples reflect astronomical intentions. Here I present the results of a survey of archaic and classical Greek temples in Sicily and compare them with temples in Greece. Using a binomial test I show strong evidence that there is a preference for solar orientations. I then speculate that differences in alignment patterns between Sicily and Greece reflect differing pressures in the expression of ethnic identity.

## Introduction

It has long been proposed that classical temples may have been aligned with respect to sunrise on certain dates. The idea was first proposed by Nissen [1] in 1869. This idea was developed further by other authors such as Penrose [2], [3], [4] and Dinsmoor, [5] who argued that a temple could be dated from its astronomical alignment. This explanation was rebutted in the 1980s by Herbert [6] on the grounds that plenty of Greek temples did not face east. Following a survey of Sicilian and southern Italian temples Aveni and Romano [7] reasserted that there is an astronomical pattern to the alignment of Greek temples, but the two most recently published statements on the subject [8], [9] both state that there was no evidence of astronomical intent. At best, there is no consensus about the answer, though a more accurate summary would be that opinion is shifting away from the notion of astronomical alignments being embedded within Greek temples.

The presence or lack of such alignments is an issue as it reflects upon Greek religious practice in the archaic (750–480 BC) and classical (480–323 BC) periods. Salt and Boutsikas [10] have proposed that Greek religious festivals, in particular Panhellenic events, may have been calibrated against the seasons using astronomical observation. Hannah [11] also observes that the observation of celestial bodies helped govern the cycles of Greek civic life. The interaction of local topography, architecture and astronomy may therefore helped shape the day-to-day functioning of a city.

A further use for the study of temple alignments is in the examination of evidence for cultural continuity across the Mediterranean. Traditionally it was thought that the Greeks colonised Sicily from the mid eighth-century onwards. [12] This has been challenged in recent years by scholars who question the degree to which it is possible to read ethnicity of settlers in the archaeological record and those who would challenge precisely what the colonisation process means.[13], [14] Greek material might be found at Sicilian sites, but this could be evidence of Greek settlement or of trade where Sicilian natives were appreciating Greek style, but using the material in a native way. The Greek temples in Sicily are clearly Greek in style, but were they used in a Greek way, or did the Sicilians use Greek architecture to build temples for local cults as the Romans did? The lack of contemporary written sources means that the exact nature of worship at some of these Sicilian sites may never be known. Yet, if it can be shown that Sicilian temples share similar astronomical alignments to Greek temples, this would contribute more evidence that these cities were practicing religion in a Hellenic style.

The results of a survey of Greek Sicilian temples presented below and a comparison with a recently published survey of Greek temples by Retallack,[9] provides a means of studying the degree of similarity of alignment of temples. The alignments provide an ‘astronomical fingerprint’, allowing a determination of what extent ‘Greek’ culture differed in the two locations.

## Materials and Methods

The survey of the Greek Sicilian temples presented here was conducted with a magnetic compass and a clinometer. Where possible temples were measured in both directions along all four sides with a compass to provide eight measurements in order to derive an axis of alignment. A clinometer used along each wall and the centre of the temple, viewing from the back to the front, provided horizon measurements to enable the calculation of the declination (astronomical latitude) that a temple faced. Frequently this was impossible, due to lack of access or enthusiastic reconstruction involving steel handrails. Therefore all measurements were also examined using the published archaeological plans, with the north arrow calibrated using local observations.

An additional problem is deciding what constitutes a meaningful independent alignment? Clearly a temple built in isolation has an axis of alignment which can be examined, but this axis emerges from the four main walls. It would not make sense to examine the astronomical alignment of each individual wall. This raises the question of whether two temples built next to each other should be counted as two independent alignments, or whether they should be thought of as sharing the same alignment.

The most obvious example would be the case(s) of what I have listed as Himera temple A/B. The later temple B was built directly over the walls of Temple A and it is likely that it was built around the physical remains rather than being independently aligned. [15] Therefore temples A and B at Himera can only be considered to be one point. At Selinous temples A and O appear to have been built at the same time as part of the same plan. Even though they sit alongside each other, it may not be reasonable to count them as two separate alignment events. [16] The same can be said for Temples F and G at Selinous. [17] Temple E which, along with temples F and G, forms a cluster of three temples sharing the same alignment has been counted as an independent alignment as it was built a century before temples F and G and therefore was laid out independently. This gives a final sample size of 41 rather than 44 points.

## Results and Discussion

To determine how many temples face east, we must first define what we mean by ‘east’. If we talk about the eastern 180° of the horizon. Then 40 out of 41 temples in the Sicilian sample face east (see data in table 1). This initially looks interesting, but then this would also be true if 40 temples had faced west. The obvious tool to analyse the result is a binomial test, which is trivial to anyone with a basic grasp of statistics, but exotic to those who work in the humanities and so is briefly described below.

**Table 1:** Alignments of Greek temples in Sicily.

	Location	Date	Deity	Az	Dec	References
**1**	Agrigento	Late 6th C	Hercules? (A)	90	0	[25], [26]
**2**	Agrigento	480	Zeus Olympios (B)	80	10	[25], [26]
**3**	Agrigento	460–440	Unknown (D)	82	8	[25], [26]
**4**	Agrigento	490–470	Athena (E)	110	−16	[25], [26]
**5**	Agrigento	450–430	Unknown (F)	87	3	[25], [26]
**6**	Agrigento	600 or 440–400	Vulcan (G)	87	2	[25], [26]
**7**	Agrigento	Hellenistic	Asklepios (H)	90	0	[25], [27]
**8**	Agrigento	470s	Demeter (I)	80	9	[22], [26]
**9**	Agrigento	Hellenistic	Demeter and Kore (L)	81	8	[26]
	Akrai	Late 6th C	Aphrodite	67	18	[28]
**10**	Camarina	Mid 5th C	Athena Pallas	107	−14	[29]
**14**	Gela	Early 6th C	Athena Lindia (A)	117	−21	[30]
**15**	Gela	6th C	Athena Lindia (B)	111	−17	[30]
**16**	Gela	5th C?	Athena Lindia (C)	114	−19	[30]
**11**	Helorus	Late 4th C	Demeter	99	−7	[31]
**12**	Heraclea Minoa	Unknown	Minos?	142	−39	[32]
**13**	Heraclea Minoa	Unknown	Aphrodite?	114	−19	[32]
**17**	Himera	7th C	Unknown (A/B)	67	18	[15]
**18**	Himera	Late 6th C or Early 5th C	Unknown (C)	67	18	[15]
**19**	Himera	480	Athena Nike	71	15	[15]
**20**	Megara Hyblaea	Early 6th C	Unknown (Ouest)	92	−2	[33], [34]
**21**	Megara Hyblaea	Early	Unknown (Heroon)	91	−1	[33], [34]
**22**	Megara Hyblaea	Third Quarter of the 7th C	Unknown (Sud)	72	14	[33], [34]
**23**	Megara Hyblaea	Last Quarter of the 7th C	Unknown (Sud à Colonnade)	77	10	[33], [34]
**24**	Megara Hyblaea	Third Quarter of the 7th C	Unknown (Nord)	95	−4	[33], [34]
**25**	Megara Hyblaea	Second half of 7th C	Unknown (Sud-Est)	86	3	[33], [34]
**26**	Naxos	610s	Aphrodite (A)	44	35	[35]
**27**	Naxos	5th C	Aphrodite (B)	61	23	[35]
**28**	Naxos	Unknown	Unknown (Tempietto)	113	−18	
**30**	Selinous	490–460	Leda and Artemis? (A/O)	96	−5	[36], [34]
**31**	Selinous	Late 6th C	Apollo (C)	96	−5	[36], [34]
**32**	Selinous	Late 6th C	Unknown (D)	96	−5	[36], [34]
**33**	Selinous	Late 7th C	Hera (E)	96	−5	[36], [34]
**34**	Selinous	Early 5th C	Unknown (F/G)	96	−5	[36], [34]
**35**	Selinous	Early 6th C	Demeter Malaphoros	83	6	[37], [34]
**36**	Selinous	Mid 6th C	Zeus Melikhios	80	8	[37], [34]
**37**	Selinous	Mid 6th C	Hekate	338	47	[37], [34]
**38**	Selinous	Mid 6th C	Unknown (M)	76	11	[38]
**39**	Syracuse	6th C	Apollo	94	−3	[39]
**40**	Syracuse	480	Athena	92	−2	[40]
**41**	Syracuse	Early 6th C	Zeus Olympios	103	−10	[41]

All dates are in years or centuries BC. The Hellenistic period is traditionally defined as the period from the death of Alexander 323 BC to the Roman conquest which would be around 212 BC in Sicily.

**Figure 1:**
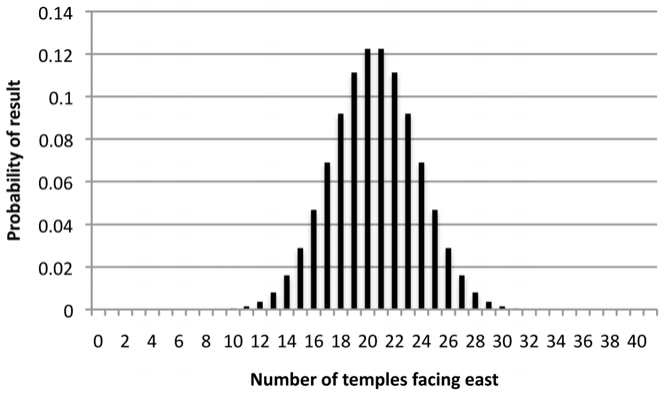
Probability of *k* temples facing east in a set of 41, if aligned randomly.

If each reader of this article were to build scale models of Greek temples and align them randomly, the average number of temples facing east would be given by *np* where *n* is the number of temples in the data set–in this case 41 and *p* is the probability between 0 and 1 that an event would happen. If the temples were aligned randomly we would typically expect 20 or 21 temples to face east. However random results vary and it would not be unusual for readers to build sets of temples with 19 or 22 temples facing east. The Binomial Distribution describes the distribution of results where there is a probability *p* that something will happen and a probability 1-*p* that it will not. The probability mass function is:
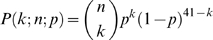



The formula above shows the chance of getting *k* successes in a sample of size *n* where there is a probability *p* of success. In the case of the temples, if they were aligned randomly, there is a 0.5 (50%) chance that a temple will face east and a 0.5 chance that it will not. The formula above is unlikely to be helpful to the typical archaeologist or classicist, but given that
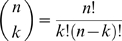
it is possible to plot the probability distribution as a graph (figure 1).

This shows that result from the survey, that 40 of 41 temples face east, is a highly improbable result to have occurred by chance. To quantify how improbable it would be helpful to calculate confidence intervals. As Ross [18] has noted these can be awkward to explain and the situation is further complicated by differing methods of dealing with confidence intervals in the social science and the mathematical sciences.

Ideally I would like to show that a distribution of temple orientations cannot be due to chance. This is impossible as there is the slightest possibility that all 41 of the temples could face the eastern half of the horizon–even if they were randomly aligned. Instead the distribution above can help me show that the probability of a result being due to chance is small.

In the social sciences it is a rule of thumb that probability distributions can be described by intervals based on the standard deviation, σ. The generalisation is that two-thirds of the time a random result will fall in a range centred on the average +/−1σ. 2σ covers 95% of random results and 3σ covers 99%. The standard deviation σ can be calculated by using the formula σ = √(*npq*). In this case it would be √(41×0.5×0.5) = 3.20. In the case of the hypothetical temple-building by readers above, this means two-thirds of readers would build between 17 and 24 temples facing east. If we expand the range to plus or minus 2σ, 14 to 27 temples, then we would account for over 95% of the readers in the experiment. 3σ would cover over 99% of cases. So by knowing the expected number of temples, and how many standard deviations away from the expected result the real result is, we can show how unusual the result is.

The method used by social scientists is an approximation of the Clopper-Pearson interval. Because of the discrete nature of the distribution this rule of thumb is quite conservative. In fact 79% of cases fall between 17 and 24 temples rather than 66%. The 2σ interval covers 97% of the distribution and the 3σ interval covers 99.85%. This may not seem drastically different to 99% but it makes the chance of a random positive result 1/690 rather than the proposed 1/100. These problems are discussed by Agresti and Couli. [19] Despite its flaws I use the social science approximation below as while this is a statistical method, the question of whether or not there is a visible preference for certain astronomical orientations in Greek temples is a social matter. Conservative estimates of significance can allow more confidence in the results.

In the case the result of 40 out of 41 temples, we are 6.09σ away from the expected result of 20.5 temples. If you're satisfied with a nominal 95% (2σ) confidence interval then any result over 20.5+2*3.20 = 26.9 is noteworthy, because only one time in twenty would an apparently significant result appear by chance. If you are a less easily convinced reader might wish to use a 99% (3σ) confidence interval 20.5+3×3.2 = 30.1 temples. This flexibility is useful because there is no commonly accepted standard of what is significant in archaeoastronomy. Indeed, it is impossible to define what is significant purely from statistics. If written documentation exists describing the use of a site then an alignment could be declared significant even if it is the only data point in a set. Likewise, throw enough tests at the data and sooner or later you will find something that passes a 2σ test. Schaefer [20] notes that 3σ results turn out to be false 50% of the time and that high σ values are desirable, especially in the absence of historical records. The value of historical records is that you can construct an argument that the measured data and the tests applied are meaningfully connected, rather than running enough tests a data set until something ‘significant’ happens. A second value of using a binomial test is that the output is quite clearly reliant upon the value of *p* which needs to be justified, and this process can make apparent any unreliable assumptions. The reader may, for example, disagree with my attempts to find a comparable sample from Retallack's survey.

Retallack [9] has recently argued that temple dedications are connected with local soil types. As part of his survey he examined the astronomical alignments of temples in Greece in order to discount their effect on dedication. Retallack surveyed 84 temples. Of these many were in a ruinous state and no meaningful alignment could be recorded, which leaves just 51 temples in the sample. This is not a major problem. The confidence in the results is derived from the sample size, not the percentage of the total number of Greek temples and 51 is a similar number to the 41 temples surveyed in Sicily. Of greater difficulty is that he only measured alignments as pointing to one of the eight major compass directions. East, northeast and southeast all face the eastern half of the horizon, and west, northwest and southwest all face the western half. It is less certain which half north-facing and south-facing temples face. I have excluded these from the survey as their easterly or westerly orientations are as unknown as for the temples with no recorded alignment. This leaves just 42 temples with clearly defined easterly or westerly orientations.

In this sample 38 temples face east and 4 face west. This is 5.24 σ away from the anticipated result of 21 temples. This is a less emphatic result than Sicilian sample, but nonetheless would support the proposal that Greek temples face east.

However ‘east’ might not mean just the eastern half of the sky. It may specifically refer to the range of the horizon that sun rises over. In Sicily this is approximately 59° to 119°. For the sake of calculation I have said that *p* is 1/6, as no temples in the Sicilian sample lie at 60° or 120°. If this is the case *np* is 41/6 = 6.83 and σ is 2.39. The number of Sicilian temples with this range is 38, which is 13σ from the expected value. Again converting Retallack's survey to a comparable sample is awkward. If we take just the temples facing the eastern eighth of the horizon, *p* is 1/8 and the expected result is 51/8 = 6.38 temples with a σ of 2.14. The actual result, 26 temples, is 8.31σ away from the expected result. Again this is lower than the Sicilian result of 13σ, nonetheless it is still a strikingly positive result.

One reason for the difference in results might be the context of their construction. Temples in Greece were frequently built upon sites that had been sacred for generations, reaching back into the Bronze Age at places like Thermon, where the later classical temples were built over the remains of Mycenaean era *megaron*. [21] There was the matter of historical tradition which meant that temples built in the archaic and classical periods might be built not only according to the cosmology of the time of construction, but also within the restraints of prior religious thought. The temples in Sicily were built in cities that, at the time of building, saw themselves as immigrants in a distant land. [22] Therefore there was no historical precedent to shape the construction of the temples. They were much more likely to be purely the products of seventh-, sixth- and fifth-century cosmology. The lack of prior foundations gave the Sicilian Greeks more freedom to express current thought in religious practice through their temples.

The self-identification of Sicilian Greeks as Greeks living overseas may have also made adherence to a Greek ideal more of an imperative to reassure both themselves and visitors from the homeland that their location made them no less Greek. It is interesting to note that Greek sanctuaries in Greece could be out in the hinterland tying territory to the city, while Sicilian temples were all built in urban or suburban sites. An ‘astronomical fingerprint’ may, along with other elements such as the architectural form and religious practice, have been part of a drive to prove the Hellenic character of a settlement. Hence, perhaps, the stronger results in Sicily than Greece. This could be testable by comparison with temple alignments in other locations like the Black Sea colonies or Hellenistic Asia. A lack of a similar adherence to astronomical orientation for temples in these regions would be a surprising result given the emphatic nature of the results in Sicily and Greece.

Indeed the extraordinarily positive results create a puzzle of their own. How is it that such a simple question, with such a definite answer, could have been discounted by many intelligent archaeologists and classicists as insignificant? The answer may be due to a failure of interdisciplinarity. In the case of Retallack's sample only half of the temples with a known direction (26 of 51) face ‘east’. The 50% failure rate perhaps makes it easy to overlook that one would only expect 1/8 of the sample, 6 or 7 temples, to fall in that range. A further issue is that the question is being asked for cultural reasons and, in the absence of historical evidence, there is no purely historical or archaeological method which can find such a pattern. Archaeologists and historians do not usually have basic statistical analysis as part of their tool-kit, and so it is possible for papers without such statistical underpinnings to appear archaeological and classical journals because the basic problems simply are not recognised. [23] Therefore research using statistical tools needs to be published either in a forum used by statistically minded scholars, or else in an open-access environment where they can access and critique the work.

At the same time while there are certainly more sophisticated tools than the binomial distribution to analysis alignment data, [24] simply throwing statistical tools at the problem or expecting the data to speak for themselves is not enough. The problem only has any relevance within its cultural context. It is therefore not enough simply to be correct, but it also has to be understandable by historians and archaeologists who, quite reasonably given their specialisms, have little knowledge of or interest in statistics. For this reason I have not used the Fisher Exact test which, while simple, is more difficult than applying a binomial test. The binomial test is a helpful bridge between the data and the interpretation as the value of *p*, as shown above, must be justified by reference to its cultural context–in this case by defining exactly what it means to say ‘east’. The simplicity of the technique also means it can be applied and disputed by anyone whose calculator has a square-root function.

Finally, expressing the results in terms of the number of standard deviations away from the expected result allows for more readings about the value of the findings than simply stating that the probability of 40 of 41 temples facing east is in excess of 1∶50,000,000,000. The probability of seeing any given sequence of three car licence plates is also billions to one, but that does not inherently make any sequence of three plates significant. It is significance in the distribution of the set, rather than the alignment of any individual temple that matters in finding evidence for an overall preference. I believe discussion above does show there was a significant preference for easterly orientations in the alignment of ancient Greek temples.
